# Removal of chromium(III) from contaminated waters using cobalt ferrite: how safe is remediated water to aquatic wildlife?

**DOI:** 10.1007/s11356-024-32741-z

**Published:** 2024-04-01

**Authors:** Joana C. Almeida, Celso E. D. Cardoso, Daniela S. Tavares, Tito Trindade, Carlos Vale, Rosa Freitas, Eduarda Pereira

**Affiliations:** 1 Chemistry Department and CICECO-Aveiro Institute of Materials, University of Aveiro, Campus de Santiago, 3810-193 Aveiro, Portugal; 2 Chemistry Department and LAQV-REQUIMTE, University of Aveiro, Campus de Santiago, 3810-193 Aveiro, Portugal; 3 Interdisciplinar Centre of Marine and Environmental Research, 4450-208 Matosinhos, Portugal; 4 Biology Department and CESAM, University of Aveiro, Campus de Santiago, 3810-193 Aveiro, Portugal

**Keywords:** Cobalt ferrite, Magnetic sorbent, Sorption, Water treatment, Oxidative stress, *Mytilus galloprovincialis*

## Abstract

The release of hazardous elements by industrial effluents to aquatic ecosystems is a potential threat to the environment. Chromium (Cr) is one of the elements whose levels in several freshwater ecosystems should be reduced to promote water reuse. In recent years, magnetic materials have gained increasing interest as sorbents because of their easy removal from treated water through magnetic separation. In this study, colloidal cobalt ferrite (CoFe_2_O_4_) particles were investigated as magnetic sorbents for chromium-aqueous chemical species. The oxidative stress responses of *Mytilus galloprovincialis* mussels exposed to 200 μg/L of Cr, resembling remediated water, were evaluated. More than 95% of Cr was removed from contaminated solutions by CoFe_2_O_4_ aqueous suspensions at pH 6 and pH 10. The kinetics of sorption experiments were examined using pseudo-1st order, pseudo-2nd order and Elovich models to evaluate which mathematical model has a better adjustment to the experimental data. The present study revealed that the levels of Cr that remained in remediated water induced limited biochemical changes in mussels, being considered safe for aquatic systems. Overall, the use of cobalt ferrite–based sorbents may constitute a promising approach to remediate contaminated water.

**Supplementary Information:**

The online version contains supplementary material available at 10.1007/s11356-024-32741-z.

## Introduction

Hazardous inorganic and organic contaminants present in industrial effluents frequently discharged into rivers, lakes, estuaries, and coastal areas should be monitored to achieve the quality criteria established by the European Water Framework Directive 2013/39/EU (Council of the European Union and Parliament 2013; ATSDR 2022).

Among the most frequently trace metals found in aquatic environments is chromium (Cr) (Ponou et al. 2011; Jin et al. 2016). This metal is commonly used in various industrial activities. For example, electroplating accounts for more than 60% of Cr release into aquatic systems, and leather tanning is the second main source of Cr followed by metallurgy and refractory (Lin 2002; Jin et al. 2016). European Pollutant Release and Transfer Register estimated those activities account for an annual discharge of 550 tons of Cr to European waters (E-PRTR 2017).

The two most common oxidation states of Cr are Cr(III), an essential nutrient in trace amounts, and Cr(VI), which is toxic and carcinogenic. A maximum total Cr concentration of 50 μg/L in drinking water is recommended by the WHO (2011), which in certain cases requires the application of water treatment procedures. Limits for wastewater and industrial effluents may exceed one or two orders of magnitude that value and varies geographically with the national regulation (Magro et al. 2012). Conventional methods for the removal of contaminants from water are chemical precipitation, ion exchange, membrane filtration, coagulation/flocculation, and electrochemical treatment (Fu and Wang 2011). These methods are relatively low cost, but are also little effective when used in realistic operational conditions. On the other hand, sorption methods have been widely used in the efficient removal of contaminants from water (Ray and Shipley 2015). The combination of magnetic properties to the sorption ability in certain materials has been of growing interest for confined environments, such as in water treatment units (Tavares et al. 2013; Mokadem et al. 2016; Zhang et al. 2016; Fernandes et al. 2017; Soares et al. 2019). Indeed, magnetic particles are available or prepared at affordable price, and, which is of uttermost importance, the sorbents in this case can be collected from waters by applying an external magnetic field. Different sorbents have already been used for Cr removal. Recently, biochar@spinel ferrite nanocomposites have been developed for Cr sorption from tannery industrial wastewater (Fito et al. 2022, 2023; Masuku et al. 2024). Fito et al. (2022) advises that the surface of spinel ferrites needs to be coated with biochar to improve the surface stability and increase the sorption capacity of pollutants. However, the synthesis of biochar-based sorbents involves pyrolysis, and the associated costs need to be addressed for scale-up. The application of spinel ferrite particles without functionalization has demonstrated potential for the removal of other metals from waters, as reported by Tavares et al. (2020). In particular, the spinel cobalt ferrite (CoFe_2_O_4_) is a promising sorbent for metals removal, due to its large surface area, contributing to its high sorption capacity. Consequently, small amount of material is required for water treatment (Kefeni et al. 2017). Also, CoFe_2_O_4_ has ferromagnetic behavior that allows its fast and easy separation from the treated water (Tatarchuk et al. 2023a, b).

Although the legislation and limits of discharge recommended are available, as well as the development of remediation approaches to avoid aquatic systems contamination, Cr can be found with concentrations, in general, between 1 and 10 μg/L in most surface waters and below 1 μg/L in groundwaters. However, according to the extent of industrial activity, Cr concentration can be higher; as it was found in surface and ground waters in the USA, with levels up to 84 μg/L and 50 μg/L, respectively (WHO 2011). Regarding Cr concentrations in estuarine and coastal areas, Ismanto et al. (2023) tracked the levels of these metals in the marine environment of Pekalongan (Indonesia) and recorded a peak value of 105.6 μg/L in the Banger River Estuary; and levels up to 10.3 μg/L were recorded in Java sea. Similar levels were reported by Belzunce et al. (2004) in the Basque coast (Basque Country), between 2 and 124 μg/L in superficial waters of the estuaries.

As a consequence of Cr presence in aquatic environments different studies described Cr bioaccumulation in aquatic wildlife (Chaâbane et al. 2020; Wei et al. 2022; Zhang et al. 2022), with concentrations measured in bivalves ranging from 0.18 to 29 mg/kg (Tapia et al. 2010). Due to its high toxicity, impacts induced by Cr in aquatic species, and in particular in bivalves, were already described in the literature, including oxidative stress in *Mytilus galloprovincialis* and *Venus verrucosa*, impacts on survival and growing rates of *Argopecten ventricosus*, immunomodulation in *M. galloprovincialis* (Sobrino-Figueroa et al. 2007; Barmo et al. 2011; Ciacci et al. 2011, 2012; Chaâbane et al. 2020).

In this context, the aim of this study is to evaluate the ability of CoFe_2_O_4_ sorbents to remove efficiently Cr species from contaminated waters in realistic conditions and to evaluate the toxicity of the concentration that remains in the treated water. Sorption kinetic experiments were performed using small mass of sorbent, low element concentrations, and complex matrices.

## Materials and methods

### Reagents

Chemicals were supplied by commercial sources and used without further purification: ammonium hydroxide solution (NH_3_ in H_2_O, 25%, Riedel-de-Häen), chromium(III) (Cr^3+^ in HNO_3_, 1000 mg/L, Inorganic Ventures), cobalt(II) chloride hexahydrate (CoCl_2_.6H_2_O, 98%, Panreac), ethanol (C_2_H_5_OH, > 99%, Panreac), humic acid sodium salt (technical grad, Sigma-Aldrich), iron(II) sulfate heptahydrate (FeSO_4_.7H_2_O, > 99%, Merk), nitric acid (HNO_3_, puriss. p.a., 65%, Merck), potassium hydroxide (KOH, > 98%, Pronolab), potassium nitrate (KNO_3_, > 99%, Merk), and sodium hydroxide (NaOH, > 98%, Pronolab).

### Synthesis of cobalt ferrite sorbents

Magnetic cobalt ferrite sorbents (CoFe_2_O_4_) were synthesized following the procedure described by Tavares et al. (2020) based on the oxidative hydrolysis of iron(II) sulfate followed by co-precipitation of Co(II) and Fe(III) ions in alkaline conditions. Ultra-pure water was first deoxygenated with N_2_ under vigorous stirring for 2 h. Then, 1.90 g (34 mmol) of KOH and 1.52 g (15 mmol) of KNO_3_ were added to 25 mL of deoxygenated water using a 250-mL round flask. This mixture was heated at 60 °C, under N_2_ and mechanically stirred at 500 rpm. After total dissolution, 10 mL of aqueous CoCl_2_.6H_2_O (6 mmol) and 15 mL of aqueous FeSO_4_.7H_2_O (11 mmol) were both added dropwise, and mechanical stirring was then set at 700 rpm. The resulting reacting mixture presented a dark-green color after complete addition of the Co(II) and Fe(II) aqueous solutions and the reaction proceeded over 30 min, after which the reaction vessel was placed in an oil bath at 90 °C, under N_2_ but without stirring, for 4 h. The resulting black powder was magnetically collected and thoroughly washed with deoxygenated water and ethanol. Finally, the particles were collected and dried in an oven at 40 °C.

### Materials characterization

The chemical and physical characterization of CoFe_2_O_4_ sorbents was performed by using various techniques.

(i) The morphology and particle size of particles were analyzed by scanning transmission electron microscopy (STEM) (Electron Microscope Hitachi, SU-70 working at 20 kV). Samples were prepared by direct deposition of an aliquot drop of an ethanolic suspension of particles over a grid of copper coated with a carbon film and then let to evaporate the solvent at room temperature.
(ii) Brunauer–Emmet–Teller (BET) surface area analyses were performed on an automated surface area analyzer (Micromeritics Gemini 2380) by means of nitrogen adsorption–desorption.
(iii) The crystalline phase in the powdered sample was identified by X-Ray diffraction (XRD) using an Empyrean PANalytical diffractometer (Cu K_α1,2_ X-radiation, *λ*_1_ = 1.54060 Å; *λ*_2_ = 1.54443 Å). The registration of the diffraction patterns was performed with a step size of 0.026°, in continuous mode, in the 15 ≤ 2θ ≤ 100° range.
(iv) Fourier transform infrared (FT-IR) spectra (in the range 4000–350 1/cm) were recorded using an attenuated total reflectance (ATR) accessory, using a Bruker Tensor 27 spectrophotometer after 256 scans with resolution of 4 1/cm.
(v) Zeta potential measurements of the colloidal samples were performed using a Zetasizer Nano ZS (Malvern Instruments). The pH of the colloid was varied between 2 and 10, either using NaOH or HNO_3_ aqueous solutions. The temperature was kept at 25 °C, and three replicate measurements of zeta potential were performed for each sample.

### Design of sorption experiments

The ability of the CoFe_2_O_4_ sorbent to remove Cr(III) from aqueous solution was evaluated by contacting a known amount of colloidal particles with an aqueous solution of Cr(III) for variable periods of time. The sorption experiments were carried out in round bottom flasks of 1000 mL at 295 ± 1 K, under mechanical stirring and at pH *ca.* 6. The solution of 2000 μg/L Cr(III) was freshly prepared by dilution of the standard commercial solution (1000 mg/L) in ultra-pure water. This concentration was selected for these studies because it corresponds to the guideline limit value for residual waters discharge in Portugal (Ministério do Ambiente 1998).

The influence of different operational conditions, such as sorbent dose, pH, ionic strength, and the presence of dissolved organic matter in the removal of Cr, was investigated.

The sorbent doses of 10, 50, 100, and 200 mg/L were tested in the removal of Cr(III) from aqueous solution at pH *ca*. 6. The influence of pH was studied at 6 and 10 for an initial Cr(III) concentration of 2000 μg/L in ultra-pure water using 50 and 100 mg/L of CoFe_2_O_4_ particles. Adjustments of pH were performed using nitric acid or sodium hydroxide solutions. Ionic strength effect on the sorption of Cr(III) was evaluated using solutions of Cr(III) prepared in ultra-pure water, mineral water, and saline water (salinity of 15 g/L). The concentration of Cr(III) tested was 2000 μg/L, at pH 6 and 10, using 50 and 100 mg/L of CoFe_2_O_4_ particles. Major constituents of the mineral water, as referred in the bottle label in mg/L, are Na^+^  = 4.4 (± 1.1); Ca^2+^  = 2.7 (± 1.6); HCO_3_^−^  = 16.5 (± 8.0) and Cl^−^  = 3.2 (± 0.9). Water pH was 5.8–7.0. Saline water was obtained by diluting filtered seawater in ultra-pure water. Humic substances were used to increase the dissolved organic matter in mineral water or saline water (salinity of 15 g/L). Sorption of Cr(III) was evaluated using sodium humic salt solutions with concentrations of 5 and 10 mg/L at pH 6 and 10, for initial Cr(III) concentration of 2000 μg/L, and 100 mg/L of CoFe_2_O_4_ particles.

In all the experiments, the start time (t_0_) was assumed the time that sorbent was first mixed with aqueous solution. After addition of particles, it was used an ultrasonic bath (~ 60 s) to obtain a better dispersion of the particles in solution. Over the aqueous Cr solution contact time with the particles, aliquots (5–10 mL) of sample were collected using Pasteur pipettes, in fixed and increasing times. These aliquots were under the influence of a magnet for approximately 15–30 min to ensure that all particles were removed. After separation of particles, the aliquots were immediately acidified (pH < 2) with 25 μL of concentrated HNO_3_ (65% v/v) and stored at 4 °C until analyzed by ICP-OES.

### Quantification of chromium, iron, and cobalt

Quantification of Cr in water samples from remediation essays was performed by inductively coupled plasma-optical emission spectometry (ICP-OES), on a Horiba Jobin Yvon Activa M spectrometer (radial configuration) equipped with a Burgener MiraMist nebulizer (sample flow of 1 mL/min), peristaltic sample delivery pump with three canals, argon flow (nebulizer: 0.02 L/min; auxiliary gas: 0.2 L/min; plasma: 12 L/min), nebulizer chamber, CCD (coupled charging device) for detection, and algorithm background correction for quantification. To ensure that the results obtained in the analysis are reliable and have the required quality, quality control was carried out in parallel with the analysis of the samples. Before starting the analysis of the samples, a calibration curve was made for which at least 5 standards of different concentrations, prepared from a mono-elemental solution was used; the curve was only accepted if a correlation coefficient of more than 0.9995 and an error of less than 10% in each standard was obtained. The intensity of the analytical signal of the sample was compared to that curve to determine the concentration. In addition, blank, certified reference material, check standard, replica of all samples, duplicate in each set of ten samples, and a recovery test in each set of ten samples were also analyzed. For the Cr quantification, the calibration curve was prepared using standards with concentrations between 4 and 2000 μg/L. It was assumed that the value of lower concentration standard (4 μg/L) is the quantification limit, and that the detection limit value is one third of the quantification limit (1.3 μg/L). The blank concentration was always under the detection limit. In the case of replica, duplicate and check standard a maximum error of 10% was accepted. The certified reference material obtained from a water interlaboratory comparison test of RELACRE entity, carried out in October 2017, was used. The range of accepting values varied between 85 and 115%. The recovery test range of accepting varied between 90 and 110%. All the results presented in this work fulfilled these quality criteria.

Iron and cobalt content in the particles were also determined by ICP-OES after acid digestion of CoFe_2_O_4_ sorbents. Briefly, about 20 mg of sample were accurately weighted into an acid-washed Teflon reactor; then 3 mL of HNO_3_ (70%) were added, and reactors were placed on a CEM MARS 5, model 240/50 microwave digestion system equipped with 12 pressurized vessels at a ramp temperature up to 140 °C for 5 min, then up to 175 °C for 3 min, and finally at 175 °C for 4 min 30 s. A second digestion cycle was carried out after addition of 3 mL of HCl (37%) for complete digestion of the materials. After digestion, samples were diluted with ultra-pure water and then analyzed by ICP-OES.

### Formulas and kinetic models

The sorption kinetics reflects the speed that the sorbate is removed by the sorbent, from the moment they come into contact (*t*_*0*_) until the moment at which the sorbent cannot retain more sorbate, i.e., the equilibration time (*t*_*e*_). Within this period, the concentration of sorbate on the sorbent material (*q*_*t*_) progressively increases as concentration in the liquid fraction (*C*_*t*_) decreases relative to the initial value (*C*_*0*_). When equilibrium is attained, the sorbate concentration on the sorbent achieves its maximum (*q*_*e*_), while the concentration of free sorbate is in its minimum (*C*_*e*_). The total amount of sorbate retained by the sorbent material in a certain time *t* (*q*_*t*_) and when equilibrium is attained (*q*_*e*_) can be determined from the following mass balance equations:

1 $${q}_{t}=\frac{({C}_{0}- {C}_{t})}{{\text{m}}}*V$$

2 $${q}_{e}=\frac{({C}_{0}- {C}_{e})}{{\text{m}}}*V$$

where *m* is the mass of sorbent existing in a given volume V of solution.

Among the various kinetic models available in the literature, the kinetic models of pseudo-1st order (or model of Lagergren) and pseudo-2nd order are two simple mathematical models, widely used. The pseudo-1st order model is expressed by the following equations:

3where *k*_*1*_ (1/h) is the rate constant of the model. This model has the disadvantage of not to adjust well to the experimental data particularly near the equilibrium (Figueira et al. 2011; Lopes et al. 2013; Tavares et al. 2014).

The pseudo-2nd order model generally adjusts better to the experimental data throughout the entire period of the sorption process (Figueira et al. 2011):

4where *k*_*2*_ (1/h) is the kinetic constant of the model (Lopes et al. 2013; Tavares et al. 2014).

Another common kinetic model is the Elovich’s model firstly used to describe the sorption of gas onto solid systems. However, more recently, this model has also been applied to the sorption processes of contaminants from aqueous solutions (Qiu et al. 2009). This model is based on the following equation:

5where *α* and *β* are, respectively, the initial sorption rate and the desorption constant.

To evaluate the effectiveness of the process, the removal percentages are calculated using the Eq. (6). The variation of ion concentration along the contact time with the sorbent material are expressed in terms of normalized concentrations (Eq. 7), which allows the comparison of the removal by various sorbates for a given sorbent, or using the same sorbate but different sorbents, regardless of the initial sorbate concentration.

6 $$\mathrm{Removal }\;(\%)=\frac{{(C}_{0}-{C}_{t})}{{C}_{0}}*100$$

7 $${C}_{t}^{\prime}=\frac{{C}_{t}}{{C}_{0}}$$

### Chromium exposure assay

After acclimation to the laboratory conditions, mussels were exposed for 28 days (according to ASTM, 2000) at 17 ± 1 °C to control (CTL, non-contaminated seawater) and Cr(III) (200 μg/L). For each treatment, three replicates were used (3-L aquaria), with six mussels in each replicate. Concentrations selected for this study are of the same order of magnitude as the Cr concentrations that remained in the remedied waters by the CoFe_2_O_4_ sorbent. After the experimental period, the mussels were individually frozen with liquid nitrogen and stored at − 80 °C, until manual homogenization of the whole soft tissue, using a mortar and a pestle under liquid nitrogen. The whole soft tissue of each organism was used with the aim to assess the general status of each specimen and not the response of a given organ or tissue. Each mussel was divided into six aliquots of 0.5 g fresh weight (FW) each for biomarkers analyses, and the remaining tissue was used for Cr quantification.

### Mussels’ biochemical responses

The biochemical endpoints assessed were (1) energy reserves, measured by glycogen (GLY) and total protein (PROT) contents; (2) oxidative stress, including antioxidant defenses measured by superoxide dismutase (SOD), catalase (CAT) and glutathione peroxidase (GPx) activities; detoxification capacity measured by carboxylesterases (CbEs) activities (CbEs-pNPA carboxylesterases activity with substrate p-nitrophenyl acetate, CbEs-pNPB carboxylesterases activity with substrate p-nitrophenyl butyrate); cellular damage assessed by lipid peroxidation (LPO) and protein carbonylation (PC) levels; and (3) neurotoxicity, evaluated as acetylcholinesterase (AChE) inhibition. A TissueLyzer II (Qiagen) set at a frequency of 10 1/s for 90 s was used for whole tissue disruption of biological samples, further homogenized the samples in the appropriate buffer for each assay at a ratio of 1:2 (w/v): except for LPO, for all the other biomarkers the potassium phosphate buffer 50 mmol/L at pH 7.0 with 1 mmol/L EDTA, 1% (v/v) Triton X-100, and 1 mmol/L DTT was used for sample extraction. To determine LPO levels, the extraction buffer was 20% (w/v) trichloroacetic acid (TCA). Samples were centrifuged at 10,000 g for 20 min at 4 °C afterwards. The supernatant was immediately collected and kept at − 80 °C if it was not used immediately. The methodology used in each biochemical assay was performed as previously described in Andrade et al. (2021).

## Results

### Major characterization of the particles

**Fig. 1:**
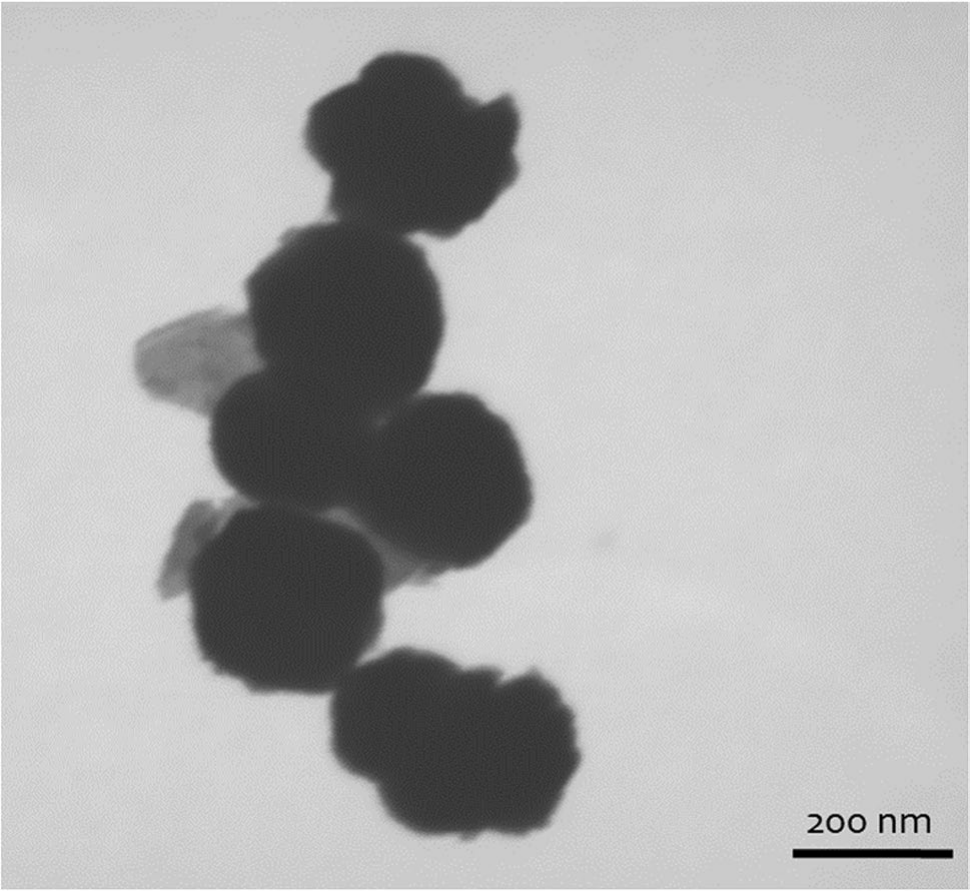
Typical STEM image of CoFe_2_O_4_ particles used as sorbents

STEM images of the CoFe_2_O_4_ samples used in this study show particles of spherical shape with an average diameter of 220 ± 20 nm (Fig. 1). The BET surface area analysis of the same samples indicates a surface area of 33.3 m^2^/g, a porosity volume of 0.07 cm^3^/g and pore diameter of 9.2 nm.

**Fig. 2:**
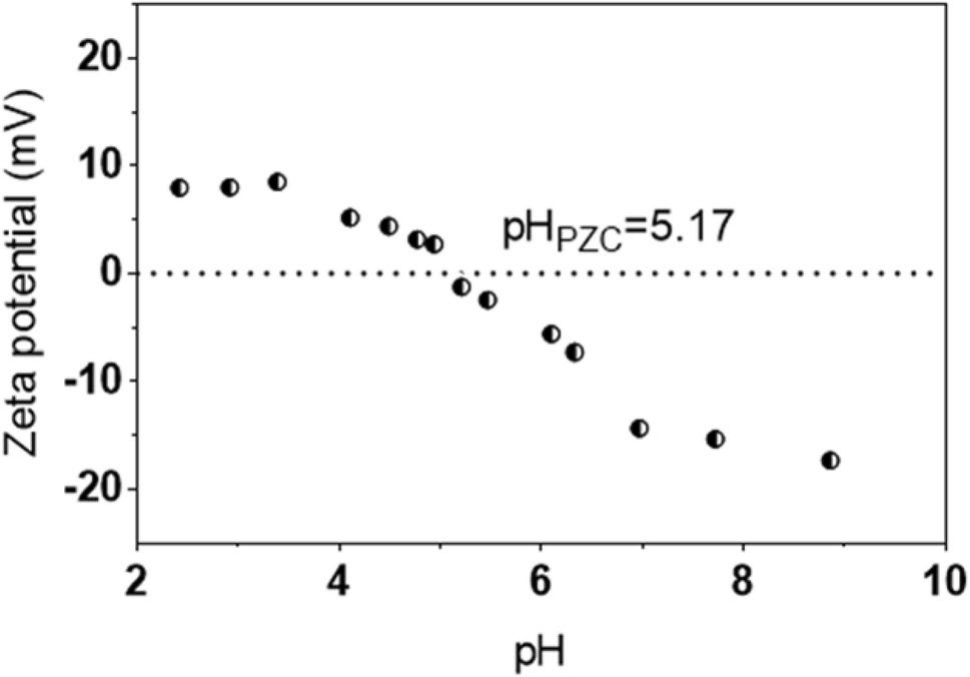
Zeta potential titration of aqueous suspensions of CoFe_2_O_4_ particles

Figure 2 displays the experimental zeta potential in function of pH for the colloid of CoFe_2_O_4_ particles. The point of zero charge (PZC) was found at about pH 5.2, meaning that particles have a negative charge distribution over the surfaces for working pH above the 5.2.

The powder XRD analysis confirmed the presence of the spinel CoFe_2_O_4_ particles (supplementary Fig. 1) and ICP measurements indicated that the Co:Fe molar ratio was 1.00:1.98, which is agreement with the stoichiometry in the molecule CoFe_2_O_4_.

Supplementary Fig. 2 shows the FTIR spectrum of a powdered sample of CoFe_2_O_4_. The band at 615 1/cm is attributed to the stretching vibration of metal–oxygen bond (Tavares et al. 2020). The broad band in the 3100–3600 1/cm region is due to OH stretching and the weak band at 1630 1/cm is due to OH_2_ bending, which indicates the presence of molecular water adsorbed to the surface (Bellusci et al. 2009; Tavares et al. 2020). The strong peak at around 1100 1/cm is due to stretching vibration of metal-OH and metal-OH_2_ bounds, which reflect the sorption of water on the oxide (Bellusci et al. 2009).

### Major factors affecting the removal of chromium from contaminated water

#### Amount of sorbent

**Fig. 3:**
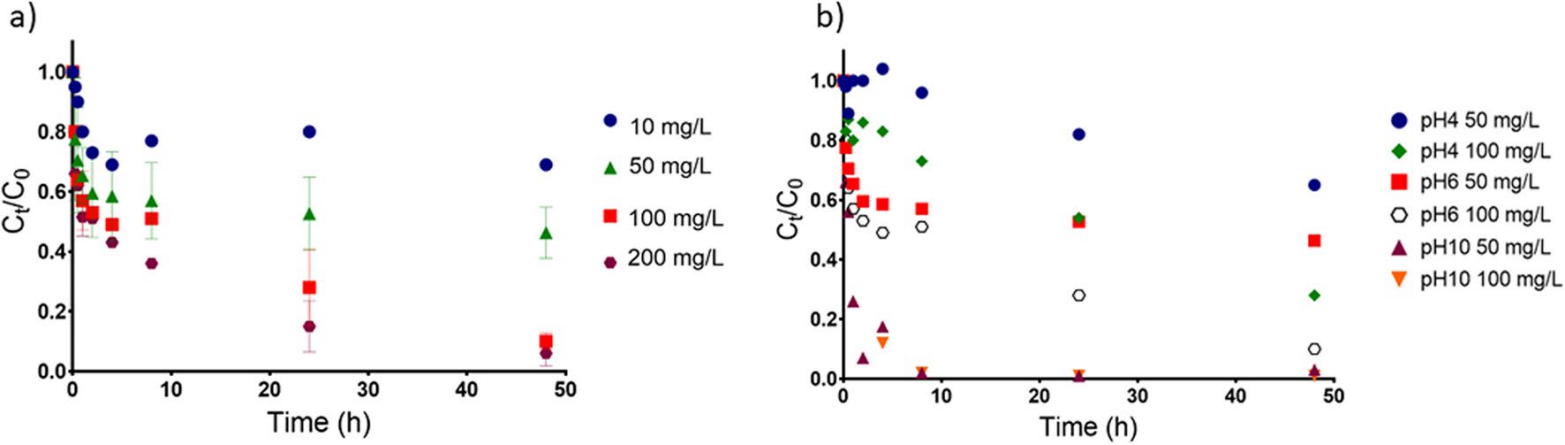
Profile of variation of the normalized concentration of Cr(III) (C_t_/C_o_)) starting with C_0_ = 2000 μg/L in ultra-pure water at room temperature, **a**) at pH 6, as function of contact time with 10, 50, 100, and 200 mg/L of CoFe_2_O_4_ particles; **b**) at pH 6 and 10, as function of contact time with 50 and 100 mg/L of CoFe_2_O_4_ particles

Figure 3a shows the normalized concentration of Cr(III) starting with *C*_0_ = 2000 μg/L in aqueous solution at time *t*(*h*) for different doses of sorbent (10, 50, 100, and 200 mg/L), at room temperature and pH at *ca.* 6. After 48 h, it was found that 50, 90, and 94% of Cr(III) was removed from water using 50, 100, and 200 mg/L of CoFe_2_O_4_, respectively.

#### pH

The influence of pH on the sorption of Cr(III) was evaluated at pH 6 and 10, using 50 and 100 mg/L of CoFe_2_O_4_ particles (Fig. 3b). The removal of Cr(III) from solution was more efficient at pH 10, reaching more than 95% of Cr(III) removal after 8 h for both 50 and 100 mg/L of sorbent dose.

#### Ionic strength

**Fig. 4:**
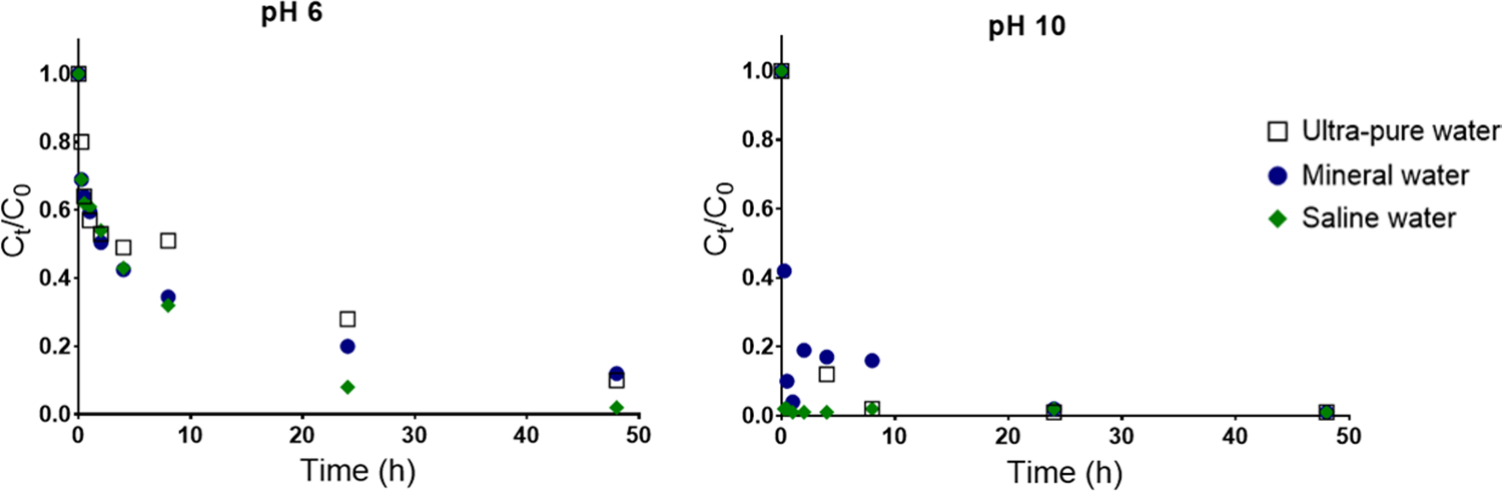
Profile of variation of the normalized concentration of Cr(III) starting with C_0_ = 2000 μg/L in aqueous solution at room temperature (mineral and saline water) at different pH (6 and 10), in function of contact time with magnetic CoFe_2_O_4_ particles (using 100 mg/L)

In order to test the capacity of CoFe_2_O_4_ particles (100 mg/L) to remove Cr(III) from complex matrices, sorption experiments were performed in mineral water and saline water (salinity 15 g/L) spiked with 2000 μg/L of Cr(III) at pH 6 and 10. The normalized concentration of Cr(III) in solution with the time is shown in Fig. 4. At pH 10, the removal of the Cr(III) present in solution was almost complete and quite fast for all the matrices. In saline water, removal exceeded 90% after 1 h. In mineral water, the efficiency decreased in all conditions. The comparison the Cr(III) sorption kinetics to the CoFe_2_O_4_ particles in ultra-pure, mineral and saline water (Fig. 4) shows that Cr(III) removal in saline water was slightly faster despite the higher amount of other ions in solution, which suggests that the addition of Cl^−^ ions to the solution favors the removal. Using 50 mg/L of sorbent efficiency only decreased slightly (presented as supplementary Fig. 3).

#### Organic matter content

**Fig. 5:**
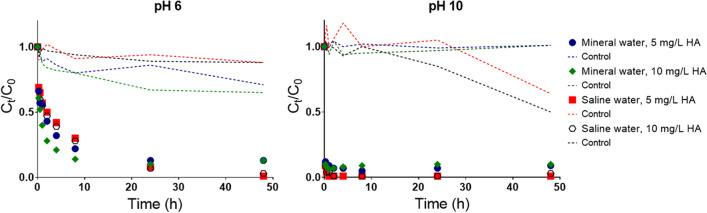
Profile of variation of the normalized concentration of Cr(III) starting with C_0_ = 2000 μg/L in mineral and saline water at room temperature with 5 and 10 mg/L of dissolved humic acids - HA) at pH 6 and 10 with time; it was used 100 mg/L of CoFe_2_O_4_ particles

The interference of dissolved organic matter in the removal of Cr(III) was tested with the addition of 5 and 10 mg/L of humic acids (HA) in mineral water and saline water. Experiments run at pH 6 and 10 with 100 mg/L of CoFe_2_O_4_ particles. The variation of the normalized concentration of Cr(III) in solution with time (Fig. 5) was similar at the two matrices and quantities of humic substances added, although being faster at pH 10. Control experiments (without addition of sorbents) showed only a slight decrease of the ratios *C*_*t*_/*C*_0_ with time at pH 6 and 10, presumably due to the formation of non-labile complexes between the Cr(III) and HA.

**Fig. 6:**
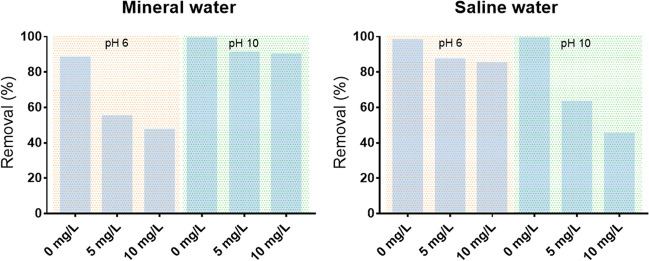
Profile of variation of the removal percentage of Cr(III) starting with C_0_ = 2000 μg/L in mineral and saline water at room temperature, without, with 5 mg/L and 10 mg/L of humic acids (HAs) using 100 mg/L of CoFe_2_O_4_ particles at pH 6 and 10

Figure 6 summarizes the Cr(III) removal percentage after 48 h for the mineral water and saline water in the presence of 0, 5, and 10 mg/L of humic acids. In mineral water, the removal of Cr was lower in the experiments with HA at pH 6, but at pH 10, the HA effect was negligible. In saline water, the presence of HA had effect on the removal at both pH although being higher at pH 10.

### Kinetic studies

**Fig. 7:**
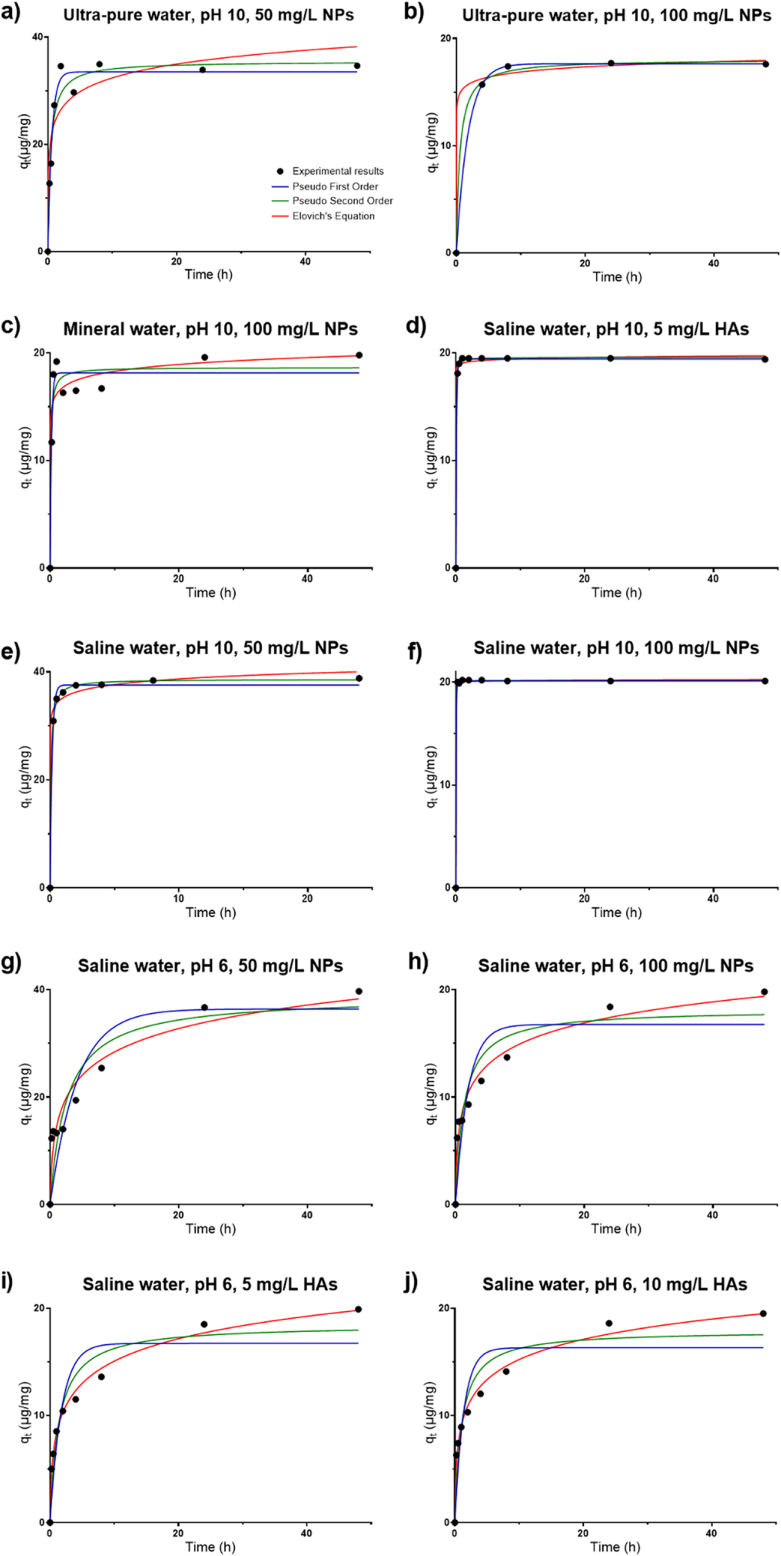
Adjustment of the kinetic models of pseudo-1st order, pseudo-2nd order and Elovich to the results obtained in Cr(III) removal test with efficiency superior to 95%, by the CoFe_2_O_4_ particles, at room temperature. Removal of Cr(III) starting with *C*_0_ = 2000 μg/L: **a**) in ultra-pure water at pH 10 using 50 mg/L of CoFe_2_O_4_ particles, **b**) in ultra-pure water at pH 10 using 100 mg/L of CoFe_2_O_4_ particles, **c**) in mineral water at pH 10 using 100 mg/L of CoFe_2_O_4_ particles, **d)** in saline water with 5-mg/L HAs at pH 10 using 100 mg/L of CoFe_2_O_4_ particles, **e**) in saline water at pH 10 using 50 mg/L of CoFe_2_O_4_ particles, **f**) in saline water at pH 10 using 100 mg/L of CoFe_2_O_4_ particles, **g**) in saline water at pH 6 using 50 mg/L of CoFe_2_O_4_ particles, **h**) in saline water at pH 6 using 100 mg/L of CoFe_2_O_4_ particles, **i)** in saline water with 5 mg/L HAs at pH 6 using 100 mg/L of CoFe_2_O_4_ particles, and **j**) in saline water with 10 mg/L HAs at pH 6 using 100 mg/L of CoFe_2_O_4_ particles

To determine which model from among those used best describes the removal process of Cr(III) by the cobalt ferrite sorbents, the graphical adjustment of the kinetic models of pseudo-1st order, pseudo-2nd order and Elovich to the experimental data (only for the best results—tests with removal percentages superior to 95%) is presented in Fig. 7.

All kinetic models used have the same number of parameters (two), implying that they have the same number of degrees of freedom; thus, the parameter *R*^2^ corresponds to a valid criterion for making a comparison of the adequacy of the various models used in adjusting the experimental results. The values obtained for the parameters of the kinetic models used in this study are presented in the Table 1.

**Table 1:** Values obtained in the adjustment of experimental results to the pseudo-1st order, pseudo-2nd order, and Elovich’s models, using the software GraphPad Prism 7

Amount of CoFe_2_O_4_ NPs	Matrix	pH	Concentration of humic acids	*q*_*e*_ exp (µg/mg)	Degrees of freedom	Pseudofirst order model				Pseudosecond order model				Elovich’s model			
						Parameters		Adjustment		Parameters		Adjustment		Parameters		Adjustment	
						*k*_1_	*q*_*e*_ (µg/mg)	*R*^2^	*s*_*y/x*_	*k*_2_	*q*_*e*_ (µg/mg)	*R*^2^	*s*_*y/x*_	α	β	*R*^2^	*s*_*y/x*_
50 mg/L	Ultra-pure water	pH 10	0 mg/L	34.7	11	1.6	33.5	0.9343	3.603	0.068	35.5	0.9299	3.722	2.307E + 03	0.2697	0.8686	5.097
100 mg/L	Ultra-pure water	pH 10	0 mg/L	17.6	3	0.55	17.6	1.000	0.04428	0.10	18.1	0.9985	0.3389	3.524E + 09	1.461	0.9961	0.5574
100 mg/L	Mineral water	pH 10	0 mg/L	19.8	7	7 4.8	18.2	0.9434	1.591	0.57	18.6	0.9230	1.855	3.957E + 07	1.087	0.9079	2.028
50 mg/L	Saline water	pH 6	0 mg/L	39.7	7	0.24	36.4	0.7828	6.287	0.010	38.8	0.8502	5.221	5.512E + 01	0.1576	0.9388	3.336
100 mg/L	Saline water	pH 6	0 mg/L	19.8	7	0.52	16.8	0.7860	3.056	0.043	18.2	0.8851	2.239	5.477E + 01	0.3525	0.9786	0.9656
50 mg/L	Saline water	pH 10	0 mg/L	38.8	6	6.6	37.6	0.9953	0.9699	0.43	38.6	0.9994	0.3328	1.951E + 10	0.6612	0.9919	1.271
100 mg/L	Saline water	pH 10	0 mg/L	20.1	7	21	20.1	0.9998	0.09877	21	20.2	0.9999	0.08530	3.422E + 160	18.59	0.9997	0.1175
100 mg/L	Saline water	pH 6	5 mg/L	19.9	7	0.56	16.7	0.8473	2.660	0.040	18.5	0.9315	1.781	4.327E + 01	0.3298	0.9933	0.5560
100 mg/L	Saline water	pH 6	10 mg/L	19.5	7	0.73	16.3	0.8153	2.819	0.054	17.9	0.9116	1.950	7.122E + 01	0.3660	0.9908	0.6278
100 mg/L	Saline water	pH 10	5 mg/L	19.4	7	11	19.4	0.9996	0.1404	2.7	19.6	0.9995	0.1469	1.826E + 46	5.683	0.9970	0.3751

### Mussel’s biochemical responses

#### Energy reserves

In terms of PROT concentrations, no significant differences were found between CTL and Cr-exposed mussels. GLY content decreased significantly in contaminated mussels (Table 2).

**Table 2:** Biochemical parameters measured in *Mytilus gallprovincialis* after 28 days of exposure to control (0 μg/L) and Cr (200 μg/L)

Biomarkers	Treatments	
	CTL	Cr
PROT	11.2 ± 0.5^a^	12.9 ± 4.6^a^
GLY	**7.2 ± 0.9**^a^	**4.9 ± 0.9**^b^
SOD	**0.08 ± 0.01**^a^	**0.21 ± 0.02**^b^
GPx	**0.13 ± 0.03**^a^	**0.27 ± 0.05**^b^
CAT	6.68 ± 1.2^a^	7.62 ± 1.1^a^
CbEs-pNPA	18.4 ± 3.4^a^	20.9 ± 2.2^a^
CbEs-pNPB	27.4 ± 6.6^a^	32.51 ± 2.97^a^
LPO	21.3 ± 4.6^a^	26.4 ± 5.3^a^
PC	1580.3 ± 91.2^a^	1973.0 ± 145.3^a^
AChE	19.4 ± 5.4^a^	20.7 ± 3.7^a^

*PROT* protein content, *GLY* glycogen content, SOD superoxide dismutase activity, GPx glutathione peroxidase activity, *CAT* catalase activity, *CbEs-pNPA *carboxylesterases activity with substrate p-nitrophenyl acetate, *CbEs-pNPB *carboxylesterases activity with substrate p-nitrophenyl butyrate, LPO lipid peroxidation levels, PC protein carbonylation levels, *AChE* acetylcholinesterase activity. Different uppercase letters (a and b) represent significant differences between the treatments (highlighted in bold).

#### Oxidative stress

The activity of SOD and GPx increased significantly in contaminated mussels compared to CTL ones, while the activity of CAT was similar regardless of the treatment (CTL vs. Cr) (Table 2). No significant differences between CTL and Cr exposed-mussels were observer both for the activity of CbEs-pNPA and CbEs-pNPB (Table 2). Levels of LPO and PC found in CTL and contaminated mussels showed no significant differences (Table 2).

#### Neurotoxicity

In terms of AChE activity, no significant differences between CTL and Cr-exposed mussels were found (Table 2).

## Discussion

### The influence of operational parameters on removal process

The present study shows the influence of the amount of sorbent, pH, ionic strength of the solution, and the presence of humic acids as a proxy of organic matter on the removal of Cr(III) from contaminated solutions by CoFe_2_O_4_ particles. All the experiments run with the initial concentration of Cr(III) of 2000 μg/L that corresponds to the guideline limit value for residual waters discharge in Portugal (Ministério do Ambiente 1998). This choice intends to give practical applicability of this study towards remediation of industrial effluents to meet environmental protection goals. For sorbent doses from 50 and 200 mg/L, the concentration of dissolved Cr decreased markedly with time, which points to the sorption of Cr(III) in solution onto the CoFe_2_O_4_ particles added to the spiked solutions. This pattern indicates that as the quantity of the sorbent material increased in water more sites were available for the Cr(III) sorption. These results are in line with several sorption studies that have achieved similar conclusion (Behnajady and Bimeghdar 2014; Shahriari et al. 2014). However, under the circumstances of the present study only for the sorbent dose of 100 and 200 mg/L, the number of sorption sites were sufficient to remove more than 90% of the amount of Cr(III) after 48 h of exposure. Shorter time may be considered if lower percentage of removal is acceptable vis-à-vis the remediation purposes. In the realist hypothesis of the initial concentration of Cr(III) in solution exceeds 2000 μg/L, as referred in the wastewater literature (Belay 2010), a previous dilution step would be necessary or the use of more sorbent to achieve the same removal efficiency.

Chromium speciation varies with the pH, as predicted by the Eh–pH diagram (Jin et al. 2016). Hence, experiments run at pH 6 and 10 to have Cr(OH)_3_ as the major species in solution. Redox potential was monitored to guarantee that according to the Eh–pH diagram Cr(III) is the only species in solution. Removal varied clearly between pH 6 and 10, and were much more favorable at pH 10, namely in the first hours. For example, after 8 h of contact between the cobalt ferrite particles and the contaminated solution removal of Cr(III) reached 95% at pH 10 and only 65% at pH 6. Taking into consideration the zeta potential measurements, the sorbent surface is enriched in ionized hydroxyl groups at pH 10, and thus originated a more negative surface charge that favored the sorption of Cr(III). In addition, at pH 10, it is expected the formation of Cr precipitates that may be associated with the sorbent negatively charged.

Despite the presence of various ions in mineral water and saline water relatively to ultrapure water, the effect on the Cr removal with a sorbent dose of 100 mg/L was minor, particularly at pH 10. The formation of chloro complexes with some metals can occur in saline water (Rocha et al. 2014). Depending on the affinity of chloro complexes to the sorbent surface, the sorption can be positive or negatively affected. In this work, the efficiency of Cr(III) removal from ultra-pure to contaminated saline water increased from 90 to > 95% at pH = 6 and remained above 95% at pH 10. It seems thus that chloro complexes promoted the removal of the Cr species superimposing the competition of ions present in solution with Cr(III) for the binding to CoFe_2_O_4_ particles.

To mimic the presence of dissolved organic matter in natural aquatic systems (Rocha et al. 2014), 5 and 10 mg/L of humic acids were added to water in the sorption experiments. Humic acids may act as strong complexing agents (Rocha et al. 2014) and have been used as sorbents for Cr(III) removal (Santosa et al. 2008). In addition, humic acids contain large numbers of acidic functional groups which, depending on pH, leads to the formation of negative charges in aqueous media (Illés and Tombácz 2004). In this way, experiments without CoFe_2_O_4_ particles were carried out and showed a predictable decrease in the concentration of Cr(III) with time. In the presence of humic acids, a fraction of Cr(III) in solution becomes unavailable to be sorbed due to the formation of stable complexes between humic acids and Cr(III) (Fig. 6). However, this effect was not observed for pH 10 in mineral water. It should be hypothesized that depending on the lability of the complex formed between Cr(III) and HA, the temperature of plasma in the ICP-OES may not be enough to quantify non-free Cr(III) ions.

In a real application, it could be necessary a pre-treatment of the effluent, in order to ensure that the pH of the effluent is between 6 and 10, and, thus, in the ambit of this work, this can be achieved by using a NaOH solution, as reported in the literature (Andrade et al. 2017).

### Kinetic studies

The sorption of metals to magnetic sorbents is a complex process and involves several mechanisms, such as adsorption on the particles surface and pores, ion-exchange, surface precipitation and complexation, and chelation (Rocha et al. 2014). Regarding the experimental results better adjusted by pseudo-1st order model, chemical sorption is the mechanism associated to the sorption process (Ho and McKay 1999), and the surface of the material is considered homogeneous, so only one binding mechanism should occur; for the studies whose sorption kinetics follows pseudo-2nd order model, the sorption is controlled by the chemisorption process involving valency forces (sharing or exchange of electrons between the sorbent and sorbate) or ionic forces (ion exchange) (Ho 2006), being that more than one binding mechanism may occur. Contrary to the models of pseudo-1st order and pseudo-2nd order, which involve certain mechanistic assumptions allowing to obtain some conclusions regarding the type of mechanism involved in adsorption processes that they model, the Elovich equation is semi-empirical and there is not a consensus regarding its nature; however, this model is usually associated to chemisorption.

Given the data presented in Table 1, it is observed that the model that best adjusts the removal of Cr(III) at pH 10 from saline water (Fig. 7e–f) is the pseudo-2nd order model, while from the other matrices (Fig. 7a–d), the results were better fit by the pseudo-1st order model. However, the *R*^2^ value obtained in the removal of Cr(III) from ultra-pure water at pH 10 (using 50 mg/L of sorbent), from mineral water at pH 10 (using 100 mg/L of sorbent) and from saline water at pH 6 (using 50 mg/L of sorbent) (Fig. 7a, c and g, respectively) is not very high, indicating that probably no kinetic models used adjusts efficiently the experimental results. On the other hand, the high value of *R*^2^ in the removal of Cr(III) from ultra-pure water at pH 10 (using 100 mg/L of sorbent) (Fig. 7b) can be due to the lack of points in the area of the graphic wherein the *q*_*t*_ increases. Therefore, it is possible to conclude that Cr(III) interacts in different ways with particles according to the present conditions (pH, matrix), but this interaction is chemical.

### Chromium sorbed on CoFe_2_O_4_ (Cr/CoFe_2_O_4_)

The FTIR-ATR spectrum of the Cr/CoFe_2_O_4_ sample shows slight changes in the bands’ intensities and a shift of *ca.* 20 1/cm in the band due to the metal–oxygen bond at 594 1/cm, as compared to the band observed at 615 1/cm in the spectrum of pristine CoFe_2_O_4_. A possible explanation involves the partial substitution of Co(II) ions by Cr(III) ions, during the removal experiments, originating a spectroscopic shift on the metal–oxygen band. This shares some similarities with the exchange mechanism proposed by Luther et al. (2013) to explain the binding of Cr(III) ions to manganese ferrite particles. In order to get more evidence, the Cr/CoFe_2_O_4_ particles were analyzed by XRD, and the results corroborate the hypothesis of cobalt sites substitution by Cr(III) ions. In the XRD diffractogram presented as supplementary Fig. 1, the most intense peaks of the spinel—the peaks corresponding to (2 2 0) and (3 1 1) planes appeared at 2θ equal to 29.81 and 35.41, respectively, and after binding of Cr(III) they appear slightly deviated to 30.08 and 35.43, respectively.

### Chromium impacts in mussels

Bivalves are benthic organisms, with long-life cycle and sedentary lifestyle, providing integrated information over time on the presence of pollutants in the environment. Furthermore, due to their ecological and economic relevance, bivalves have been also widely used in laboratory studies to assess the impacts of pollutants (for review, Strehse and Maser (2020)). For this reason, the mussel species *Mytilus galloprovincialis* was used in the present study to assess the toxic impacts induced by Cr. The results obtained demonstrated that the PROT content was not altered after exposure to Cr while the GLY concentration decreased in Cr-exposed mussels, which may indicate that in the presence of the metal mussels were using this energy reserve, most probably to fuel up the activation of defense mechanisms. In fact, the activity of the antioxidant enzymes SOD and GPx was enhanced in contaminated mussels, indicating the response of organisms to a possible overproduction of reactive oxygen species (ROS) generated by the presence of Cr. Nevertheless, CAT activity was not altered, which could result from the fact that GPx and CAT are both responsible for the elimination of the same ROS (H_2_O_2_). Previous studies conducted by Chaâbane et al. (2020) revealed similar results in the clam *Venus verrucosa* exposed to Cr(VI) (1, 10, and 100 μg/L), with an induction of enzymatic antioxidant activities (SOD, GPx).

In what regards to detoxification capacity, the results obtained demonstrated that CbEs were not involved in Cr elimination, with similar activity in CTL and Cr-exposed mussels. CbEs are commonly involved in pesticides detoxification in bivalves (Antognelli et al. 2006; Iummato et al. 2018), butand the study conducted by Cunha et al. (2022) also demonstrated the lack of activation in the presence of the rare earth element Lanthanum (La). Nevertheless, Chaâbane et al. (2020) demonstrated the induction of phase II detoxification enzymes GSTs, in *Venus verrucosa* exposed to 10 and 100 μg/L of Cr(VI), which may indicate higher toxicity of Cr hexavalent form in comparison to trivalent one (Zhou et al. 2017).

Although no detoxification was observed in contaminated mussels, because of increased antioxidant defense capacity, and, at the same time, low metal toxicity, mussels exposed to Cr revealed no cellular damage. Evaluating the impacts of Cr(VI), Chaâbane et al. (2020) demonstrated increased MDA content in the gills and digestive gland of *V. verrucosa*. Also, Emmanouil et al. (2007) showed that the mussel species *Mytilus edulis* exposed to 10 and 200 μg/L of Cr(VI) revealed increased MDA content after 10 days exposed to 200 μg/L of Cr(VI).

Accompanying the lack of cellular damage, mussels also revealed no neurotoxic effects in the presence of Cr. Nevertheless, several metals already demonstrated to cause neurotoxicity in bivalves, even at lower concentrations such as the case of Ti that at concentrations of 280 μg/L showed neurotoxic impacts in the mussel species *M. galloprovincialis* (Sureda et al. 2018). Also, Pinto et al. (2019) demonstrated that La caused neurotoxicity in bivalves at concentrations ranging from 0.1 to 10 mg/L La. The lack of neurotoxicity observed in the present study might indicate the low toxicity of Cr (III) on mussels AChE activity.

## Conclusion

For all the studied experimental conditions, only 100 mg/L of cobalt ferrite particles proved to be a very efficient material in the removal of Cr(III) from contaminated waters of different complexity. At pH 6 and 10, while chloro complexes promote the removal of Cr specie, humic acids are chelators and compete with cobalt ferrite particles for Cr removal. Chromium concentration in remediated water induced limited alterations on mussels’ oxidative stress, and no neurotoxicity was observed, highlighting the capacity of this approach to produce decontaminated water safe to aquatic wildlife.

Advantages of cobalt ferrite sorbents in water treatment are ease and reproducibility of synthesis, rapid removal of waters by magnetic separation, generation of low amounts of solid waste, possibility of reuse, which might be relevant for example for analytical preconcentration methods (Giakisikli and Anthemidis 2013). However, further studies are recommended such as the assessment of the impact of these materials and their long-term stability, namely due to the cobalt release to the treated waters. Lastly, the behavior of these materials has yet to be tested for the total Cr removal from real wastewaters.

### Supplementary Information

Below is the link to the electronic supplementary material.

Supplementary file1 (DOCX 345 KB)
