# Supplementary material for: Removal of chromium(III) from contaminated waters using cobalt ferrite: how safe is remediated water to aquatic wildlife?
Source: Environ Sci Pollut Res Int. 2024 Apr 1;31(19):28789–802. doi: 10.1007/s11356-024-32741-z (PMC11058620; doi:10.1007/s11356-024-32741-z)
Supplement: Supplementary file 1 — Supplementary file1 (DOCX 345 KB) [file 11356_2024_32741_MOESM1_ESM.docx]

**Supplementary information**

**Figure 1**


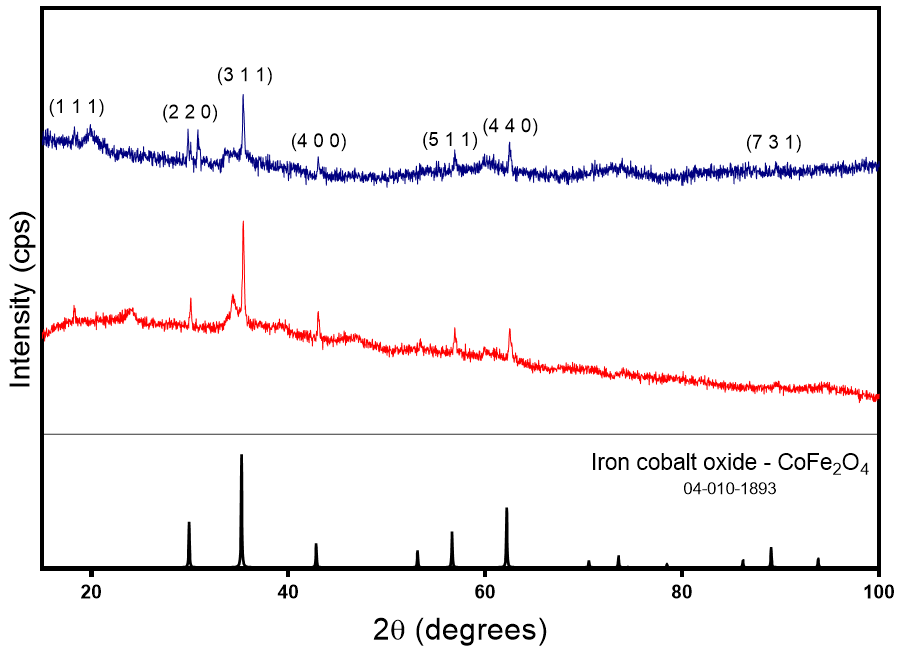


**Fig. 1.** Powder XDR patterns of the CoFe_2_O_4_ (blue line) and Cr/CoFe_2_O_4_ (red line) samples.

**Figure 2**


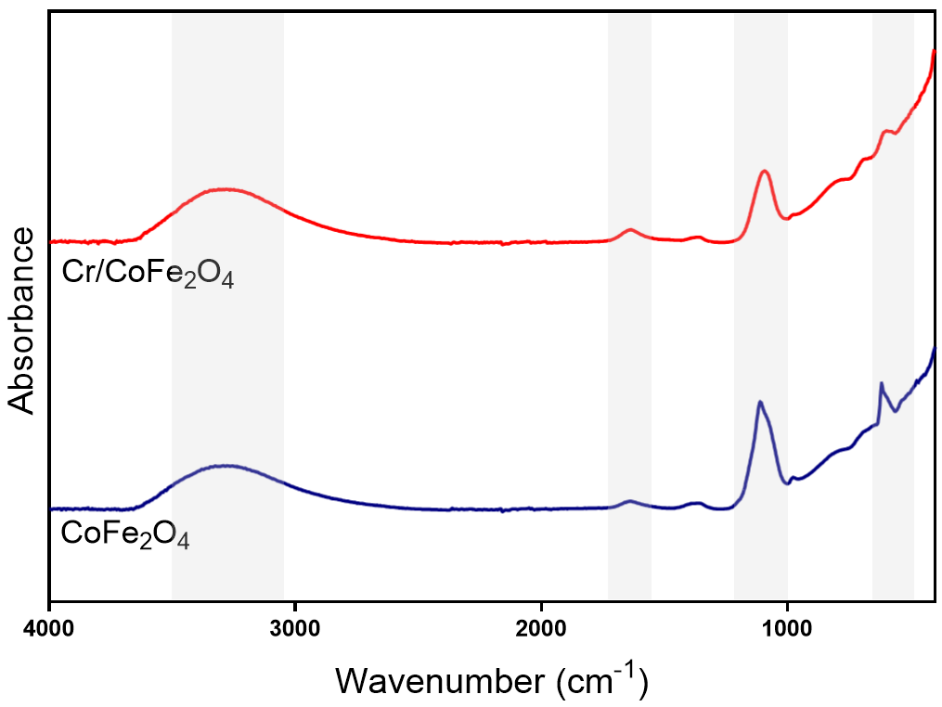


**Fig. 2.** FTIR-ATR spectra of CoFe_2_O_4_ and Cr/CoFe_2_O_4_ particles used for Cr(III) removal from ultra-pure water.

**Figure 3**

**Fig. 3.** Profile of variation of the normalized concentration of Cr(III) starting with C_0_=2000 µg/L in aqueous solution at room temperature (mineral and saline water) at different pH (6 and 10), in function of contact time with magnetic CoFe_2_O_4_ NPs (using 50 mg/L).
